# High accuracy epidermal growth factor receptor mutation prediction via histopathological deep learning

**DOI:** 10.1186/s12890-023-02537-x

**Published:** 2023-07-05

**Authors:** Dan Zhao, Yanli Zhao, Sen He, Zichen Liu, Kun Li, Lili Zhang, Xiaojun Zhang, Shuhao Wang, Nanying Che, Mulan Jin

**Affiliations:** 1 Department of Pathology, Beijing Chest Hospital, Capital Medical University/Beijing Tuberculosis and Thoracic Tumor Research Institute, Beijing, 101149 China; 2 Digital Manufacturing Laboratory, Beijing Institute of Technology, Beijing, 100081 China; 3 Thorough Lab, Thorough Future, Beijing, 100036 China; 4 Department of Pathology, Beijing Chaoyang Hospital, Capital Medical University, Beijing, 100020 China

## Abstract

**Background:**

The detection of epidermal growth factor receptor (EGFR) mutations in patients with non-small cell lung cancer is critical for tyrosine kinase inhibitor therapy. EGFR detection requires tissue samples, which are difficult to obtain in some patients, costing them the opportunity for further treatment. To realize EGFR mutation prediction without molecular detection, we aimed to build a high-accuracy deep learning model with only haematoxylin and eosin (H&E)-stained slides.

**Methods:**

We collected 326 H&E-stained non-small cell lung cancer slides from Beijing Chest Hospital, China, and used 226 slides (88 with EGFR mutations) for model training. The remaining 100 images (50 with EGFR mutations) were used for testing. We trained a convolutional neural network based on ResNet-50 to classify EGFR mutation status on the slide level.

**Results:**

The sensitivity and specificity of the model were 76% and 74%, respectively, with an area under the curve of 0.82. When applying the double-threshold approach, 33% of the patients could be predicted by the deep learning model as EGFR positive or negative with a sensitivity and specificity of 100.0% and 87.5%. The remaining 67% of the patients got an uncertain result and will be recommenced to perform further examination. By incorporating adenocarcinoma subtype information, we achieved 100% sensitivity in predicting EGFR mutations in 37.3% of adenocarcinoma patients.

**Conclusions:**

Our study demonstrates the potential of a deep learning-based EGFR mutation prediction model for rapid and cost-effective pre-screening. It could serve as a high-accuracy complement to current molecular detection methods and provide treatment opportunities for non-small cell lung cancer patients from whom limited samples are available.

## Background

According to GLOBOCAN’s Global Cancer Statistics in 2018, lung cancer is the leading cause of cancer morbidity and mortality worldwide [1], and 85%-90% of them are non-small cell lung cancers (NSCLCs). Targeted therapy is an effective treatment method for NSCLC [2]. It requires the patient’s gene mutation status, such as the presence of an epidermal growth factor receptor (EGFR) mutation, to be determined by performing polymerase chain reaction (PCR) or next-generation sequencing (NGS). Due to sample limits, some patients cannot be tested and thus are not able to receive targeted therapy.

Developments in artificial intelligence have revealed the applicability of deep learning in various fields, including image classification and segmentation [3]. In recent years, researchers have successfully developed several medical diagnostic systems [4–9]. In the field of histopathological diagnosis, researchers have achieved promising results for malignant tumour detection in whole-slide images (WSIs) of lung [10], gastric [11], colon [12], prostate [13–18], and lymph node tissues [19–22], among others.

Unlike tumour detection, for which regions of interest can be annotated, EGFR mutation prediction has only slide-level information, presenting a weakly supervised learning scenario [23–25]. In a recent study, a patch-level EGFR mutation prediction model was developed for adenocarcinoma (ADC) with a high patch-level area under the curve (AUC) on a test set containing frozen formalin-fixed paraffin-embedded tissues and biopsies [10].

To further boost the clinical significance of EGFR mutation prediction for both ADC and squamous cell carcinomas (SCC), we aimed to develop a deep learning model with high accuracy. The model is intended to provide an accurate and cost-effective alternative to molecular detection methods, particularly for patients with limited tissue samples. To ensure the efficacy of the deep learning model with slide-level information, we designed it to concentrate on cancerous area at the pixel level using the NSCLC diagnostic model proposed in our previous work [26]. Moreover, we proposed a double-threshold approach to improve the applicability of the model by categorizing NSCLC cases into EGFR-positive, EGFR-negative, and EGFR-uncertain groups. Meanwhile, by incorporating the ADC subtype information, the model achieved superior sensitivity and specificity.

## Methods

### Tissue specimens

A total of 326 haematoxylin and eosin (H&E)-stained slides, including 37 lung SCCs and 289 ADCs, were collected from Beijing Chest Hospital, China. The samples contained 121 biopsies and 205 surgical sections (from lobectomy, segmental, and wedge resection surgeries). Traditional EGFR mutation status diagnosis was made by pathologists according to WHO guidelines using PCR or NGS, resulting in 138 positive and 188 negative cases, as the gold standard. The EGFR mutations considered in this study included L858R, 19Del, G719X, and L861Q.

### Deep learning model

In a previous work, we developed a diagnostic model based on DeepLab v3 for NSCLC with a slide-level AUC of 0.988. Taking a WSI as the input, the deep learning model automatically outputs the NSCLC areas at the pixel level.

Unlike the supervised learning cancer detection model, in which pixel-level annotations of the cancerous areas were known in the training stage, the EGFR mutation prediction displayed a weakly supervised scenario. Specifically, the only supervised information available consisted of slide-level labels (positive or negative). Regarding pathology, the positive cases were WSIs with some regions containing an EGFR mutation but with the exact location unknown. The only prior knowledge available for the model was that the EGFR-mutant regions were malignant tumours.

**Fig. 1:**
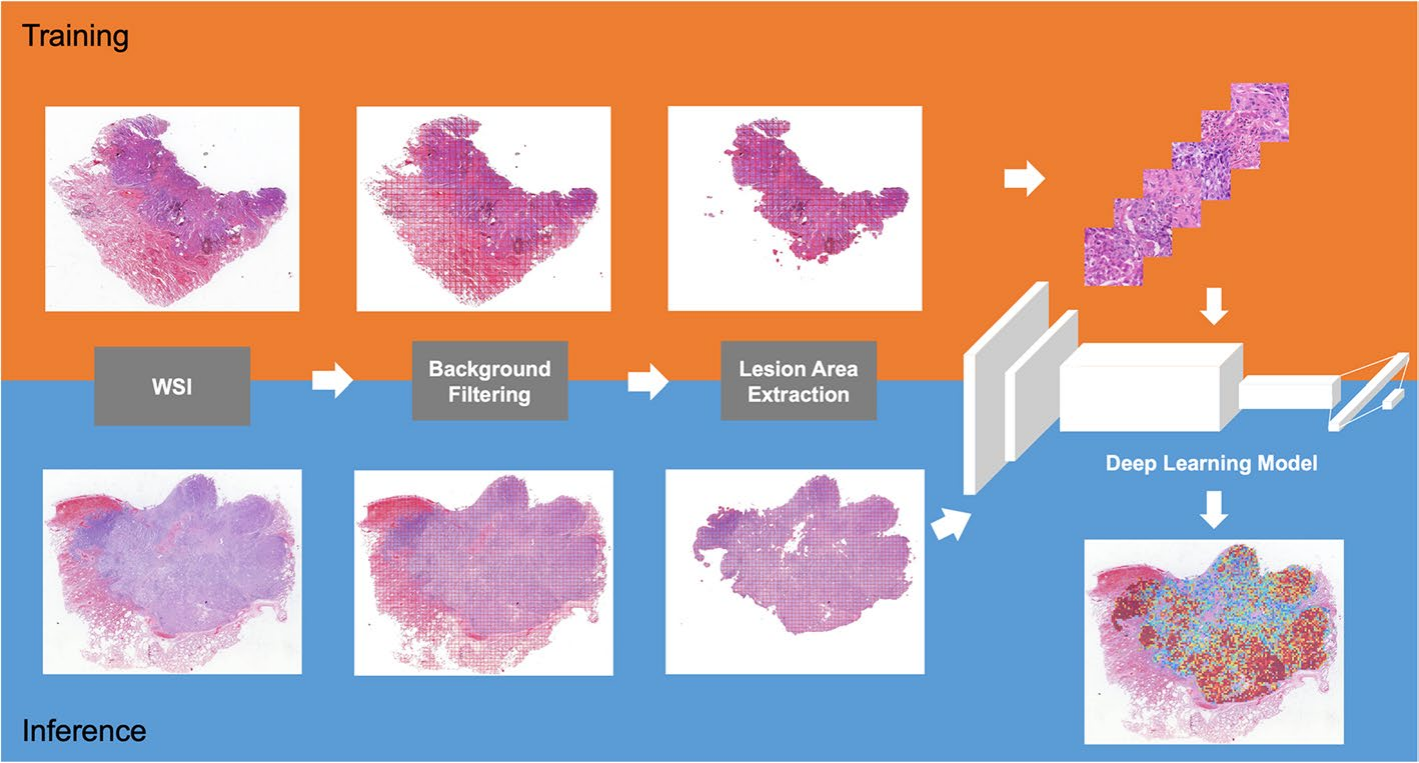
The framework of this study

We randomly selected 226 NSCLC WSIs (positive: 88, negative: 138; ADC: 210, SCC: 16) as the training set and divided them into patches with 320$\times$320 pixels at 200$\times$. As illustrated in Fig. 1, we first input the patches into the NSCLC diagnosis model and identified the pixel-level cancerous areas. Next, in order to train the EGFR mutation prediction model, we assigned the slide-level label related to EGFR mutation status directly to the patches derived from the corresponding slide. In practice, to make the model concentrate on the pixel-level cancerous area, we selected all the patches containing cancers and assigned them with slide-level mutation status labels. The patches were then input into the classification model (ResNet-50) in a supervised manner. The deep learning model was trained from scratch. Data augmentation, which included random rotation, gaussian and motion blurs, color jittering in brightness (0.0-0.2), saturation (0.0-0.25), contrast (0.0-0.2), and hue (0.0-0.04), was performed during training.

The ResNet-50 model was trained and evaluated on an Ubuntu server with four Nvidia GTX1080Ti GPUs using TensorFlow. The Adam optimizer with a fixed learning rate of 0.001 was used. The batch size was set to 80 (20 per GPU), and training was terminated after 20 epochs.

The test set included 100 NSCLC slides (positive: 50, negative: 50; ADC: 87, SCC: 13). Patch-level EGFR mutation probability heatmaps were derived using the trained classification model. The slide-level probability was obtained by averaging all the patch-level predictions.

### Double-threshold approach

If a medical case is not readily categorized as positive or negative during the diagnostic process, pathologists can actively designate the case as uncertain. Therefore, when making decisions, the human brain specifically identifies a *gray area* for uncertain things.

The deep learning model in this study provides a probabilistic prediction. The closer the result is to 1, the more the model leans towards a positive outcome, and vice versa for a negative outcome. In previous research on deep learning, a threshold value was given. If the probability was greater than the threshold, the prediction was positive, and if the probability was lower, the prediction was negative. This is called a *single-threshold strategy*. This seemingly strict strategy does not differentiate between samples of varying difficulty.

We proposed a *double-threshold strategy* that differs from the traditional single-threshold strategy. In our approach, we defined two thresholds: *M* and *m* ($M > m$). Cases whose probabilities were greater than *M* or less than *m* were treated as positive or negative samples. Cases with probabilities between *M* and *m* could be further diagnosed using PCR or NGS. When the double-threshold strategy was applied, the sensitivity and specificity were derived using near-certain cases, i.e., cases with a probability greater than *M* or less than *m*. Two thresholds were chosen on the premise of near 100% sensitivity and near 90% specificity. The double-threshold strategy not only simulates the uncertain situation that people could not distinguish or recognize naturally but also stems from our insights into the application of artificial intelligence.

### Evaluation metrics

We mainly used sensitivity, specificity, and accuracy to evaluate the model performance. These metrics were defined as follows: Sensitivity = TP / (TP + FN); Specificity = TN / (TN + FP); Accuracy = (TP + TN) / (TP + FN + FP + TN); where TP, TN, FP, and TN represent the true positive, true negative, false positive, and false negative, respectively.

We also adopted the following metrics in this study: Positive Rate = (TP + FP) / (TP + FN + FP + TN); Positive Predict Value (PPV) = TP / (TP + FP); Negative Predict Value (NPV) = TN / (TN + FN); False Negative Rate = 1 - Sensitivity; False Positive Rate=1 - Specificity.

The receiver operating characteristic (ROC) curve was plotted using the matplotlib package in Python, in which the abscissa was 1 - Specificity and the ordinate was Sensitivity. The AUC was defined as the area under the ROC curve; a large AUC meant improved predictive accuracy. In addition, we adopted $\chi ^2$ analysis to measure whether there was a significant difference for a given hypothesis. We created Python scripts to calculate $\chi ^2$ and the *P* value. If $P < 0.05$, a significant statistical difference was confirmed.

## Results

### Model performance

**Fig. 2:**
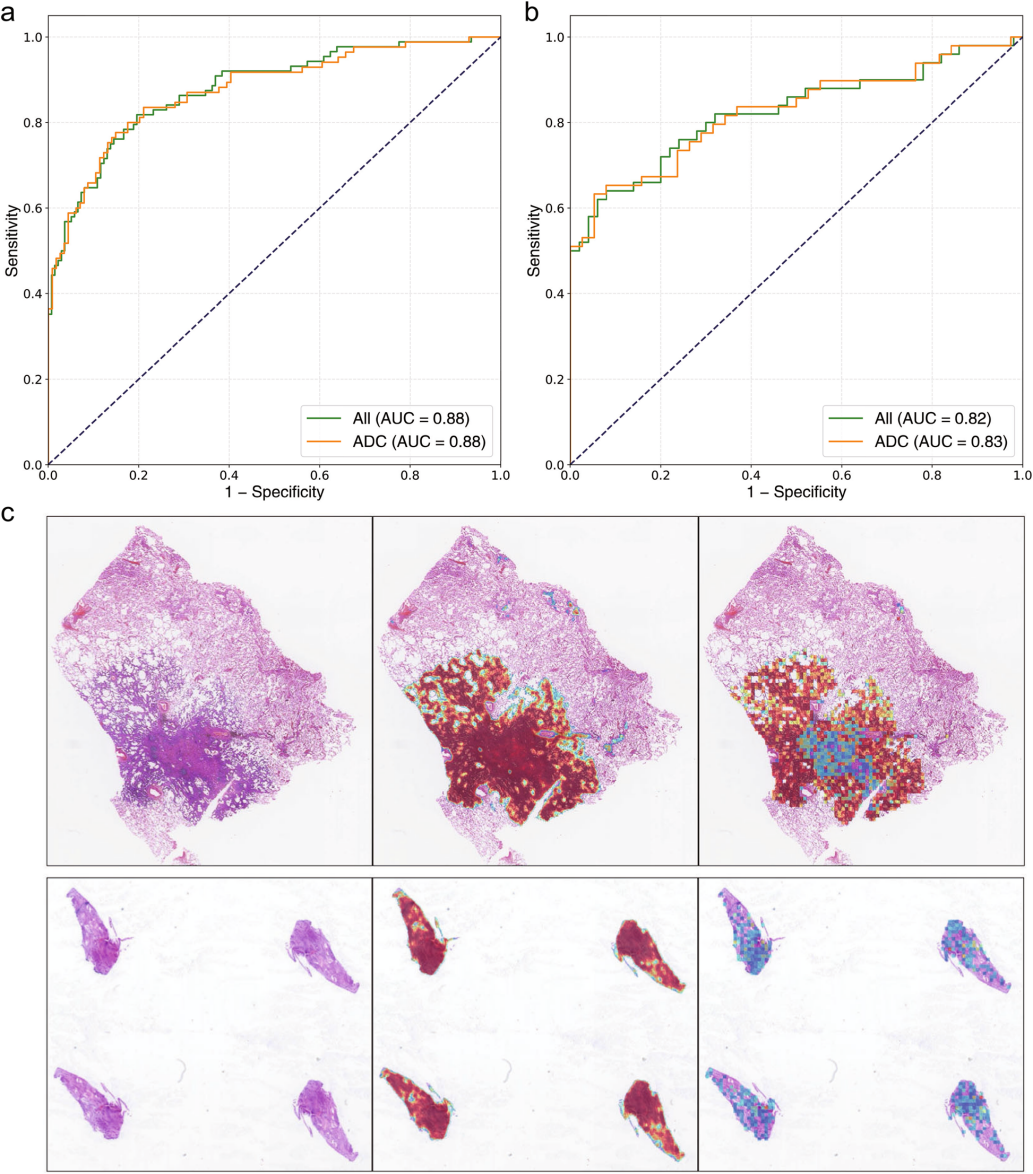
Model performance. **a** ROC curve of the model performance on the training set. **b** ROC curve of the model performance on the test set. **c** Two examples for the WSI, cancer prediction heatmap, and EGFR mutation prediction heatmap. The upper case is an ADC with the main subtype as lepidic, and infiltrating area with a few tumor cells as acinar. The EGFR mutation prediction well reflected this feature. The lower case is an SCC biopsy with a true negative prediction

The detailed model performance is described in detail in Table 1. Figure 2a gives the slide-level EGFR mutation prediction ROC curve on the training set. Figure 2b shows the ROC curve on the test set with a slide-level AUC of 0.82. By fixing the threshold as 0.36, we derived the optimal evaluation metrics of 76% sensitivity and 74% specificity. A threshold of 0.36 indicated that all WSIs whose prediction probabilities were greater than 0.36 were considered to contain EGFR mutations.

**Table 1:** Test data distribution and model performance

		Number	TP	FN	FP	TN	Positive Rate	Sensitivity	Specificity	PPV	NPV	Accurracy
Subtype	ADC	88	38	11	12	27	56.8%	77.6%	69.2%	76.0%	71.1%	73.9%
	SCC	12	0	1	1	10	8.3%	0.0%	90.9%	0.0%	90.9%	83.3%
Specimen	Surgical	70	31	7	7	25	54.3%	81.6%	78.1%	81.6%	78.1%	80.0
	Biopsy	30	7	5	6	12	43.3%	58.3%	66.7%	53.8%	70.6%	63.3%
Total		100	38	12	13	37	51.0%	76.0%	74.0%	74.5%	75.5%	75.0%

Figure 2c shows the predicted results from a surgical ADC and a biopsy lung SCC. The left, middle and right subfigures are WSIs, cancer detection, and EGFR prediction heatmaps, respectively. The heatmaps are intuitive representations with which pathologists can accurately locate the specific regions correlated to EGFR mutation. The main subtype of the ADC case was lepidic, with the infiltrating area containing a few acinar tumour cells. The heatmap in the lepidic area is dark red, indicating a high EGFR mutation rate. However, most of the infiltrating foci were fibrotic stroma with only a few acinar adenocarcinoma cells, and the EGFR mutation possibility was low (heatmap in blue). The SCC biopsy sample revealed a true negative prediction.

### Double-threshold approach

**Fig. 3:**
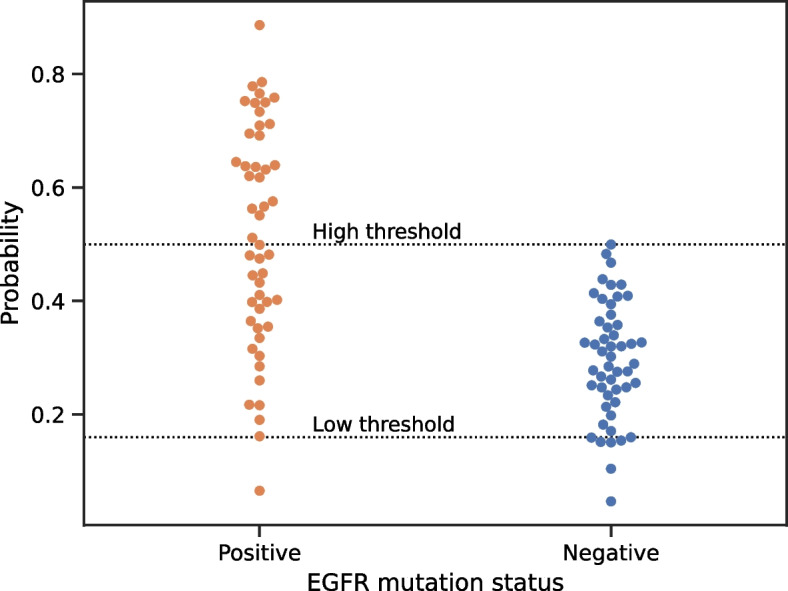
Dot chart of the EGFR mutation predictions for all the cases

For the entire NSCLC test set, we set the double thresholds to 0.50 and 0.16 and achieved a sensitivity of 100.0% and a specificity of 87.5% (as plotted in Fig. 3).

This means that for cases with values higher than 0.50, the false-positive rate was 0, and for cases with values lower than 0.16, the false-negative rate was 12.5%. After stratification, 67% of the cases lay between the two thresholds, which needed to be confirmed by PCR/NGS. That is, the NSCLC EGFR mutation status could be determined by H&E-stained slides with high accuracy in 33% of cases.

### Subtype information

In accordance with WHO guidelines [27], we further divided the ADC test set into five main subtypes: lepidic, papillary, micropapillary, acinar, and solid. A total of 83 cases of ADC were included; 5 cases were excluded (the dominant subtype of 3 cases could not be determined, and 2 cases were invasive mucinous adenocarcinoma, which is a special subtype of ADC).

By providing the ADC subtype information, the model performance further improved. The performance on different subtypes of ADC is shown in Table 2. In terms of the $\chi ^2$ test, it was evident that the performance of the model on papillary subtypes was higher than on nonpapillary ($P < 0.05$) subtypes; the performance was lower on solid subtypes than on nonsolid ($P < 0.01$) subtypes in terms of EGFR-positive rates.

**Table 2:** Performance of our model on different subtypes of ADC. The $\chi ^2$ is computed by comparing with other subtypes of ADC

Subtype	Number	TP	FN	FP	TN	Positive Rate	Sensitivity	Specificity	Accurracy	$\chi ^2$	*P*-value
Lepidic	1	1	0	0	0	100%	100%	-	100%	-	-
Papillary	27	21	3	0	3	77.8%	87.5%	100%	88.9%	6.53	0.01
Micropapillary	8	4	3	0	1	50%	57.1%	100	62.5%	0.223	0.64
Acinar	28	11	3	7	7	64.3%	78.6%	50%	64.3%	0.722	0.40
Solid	19	1	1	3	14	21.1%	50%	82.4%	78.9%	13.67	0.00
Total	83	38	10	10	25	57.8%	79.2%	71.4%	75.9%	-	-

Because of the clinical value of every subtype, this study introduced subtypes into the EGFR “double-threshold” screening model.

**Fig. 4:**
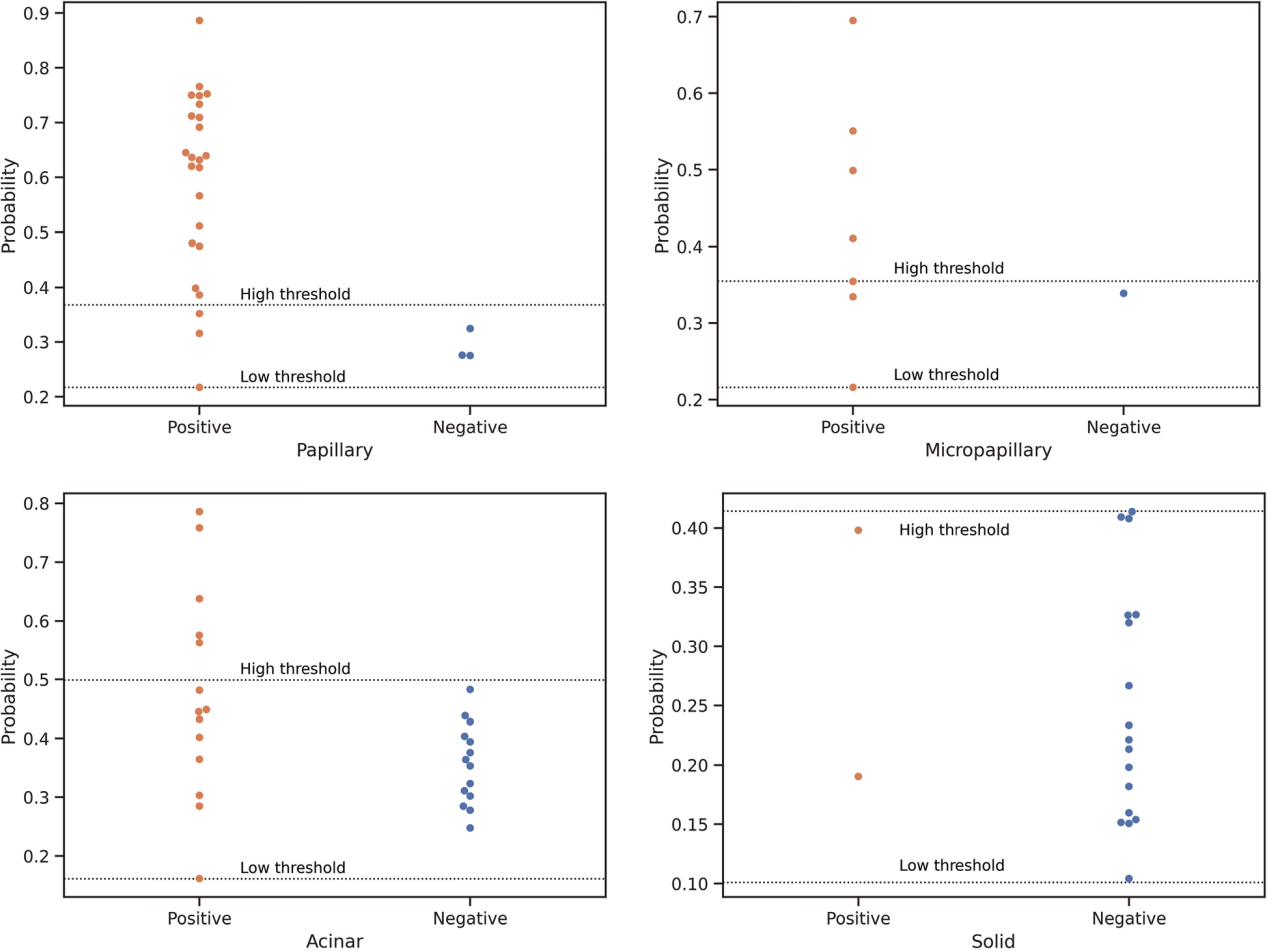
Dot charts of model predictions for cases with different ADC subtypes

For the purpose of 100% sensitivity, as plotted in Fig. 4, we set double thresholds for the acinar (0.161 and 0.499), papillary (0.217 and 0.386), micropapillary (0.216 and 0.355) and solid types (0.104 and 0.414). Since there was only one case of the adherent type, we excluded it.

The sum of positive cases (above the upper threshold) and negative cases (below the lower threshold) was the “screening number”, and the ratio of the screening number to original number was the “screening rate”.

The model performance is shown in detail in Table 3. The screening rates were 77.8%, 50%, 17.9% and 0, respectively. The screening rate of ADC (including adherent type) was 37.3%. This means that 37.3% of patients with ADC can be predicted with high accuracy. This is especially helpful for patients with advanced ADC who do not have sufficient tissue samples.

**Table 3:** Model performance on different ADC subtypes with the double-threshold approach

Subtype	Number	Low threshold	High threshold	Screening population	Screening rate
Lepidic	1	-	-	1	100%
Papillary	27	0.217	0.386	21	77.8%
Micropapillary	8	0.216	0.355	4	50%
Acinar	28	0.161	0.499	5	17.9%
Solid	19	0.104	0.414	0	0
Total	83			31	

### Prediction visualization

**Fig. 5:**
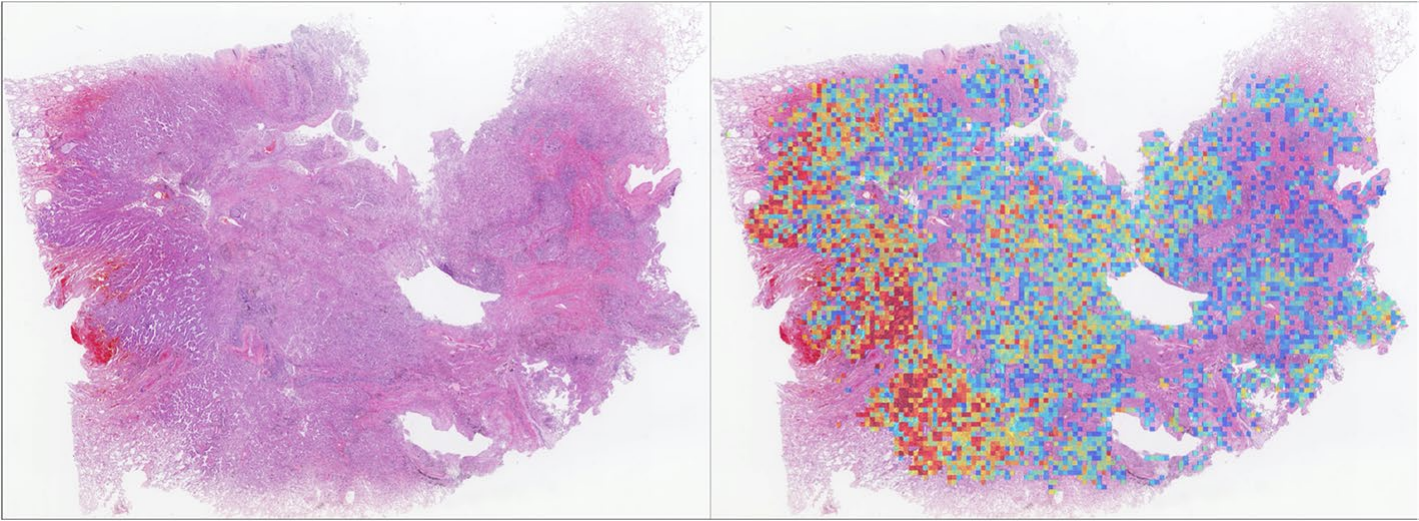
ADC case with small mutational regions. In the H&E-stained slide, we find most of the ADC to be solid; only the left side area has lepidic and papillary subtypes. The dark red regions in the predicted heatmap appear to be lepidic and papillary. Due to the low proportion, the probability is 0.35, lower than the threshold to be positive

Figure 5 shows a false negative case of the EGFR mutation prediction. H&E-staining showed that most of this case was solid type, and a small part in left area contained lepidic and papillary types.

The dark red in the heatmap is mainly located on the left side in areas of the adherent type and papillary type; the solid-type area on the right is negative. Because of the small area of the positive region, the average result of the whole section was lower than the positive threshold, which led to a false negative.

Although this case was judged as a false negative, this error may not affect clinical decisions. Because the heatmap is intuitive, if there is heterogeneity, the pathologist can make the corresponding judgement on the basis of pathological knowledge and pay sufficient attention to that case.

## Discussion

In this research, we built a deep learning model for EGFR mutation prediction with an AUC of 0.82 (sensitivity: 76%, specificity: 74%). Using the double-threshold approach, patients with NSCLC could be classified as EGFR positive, EGFR negative, or unclear. The EGFR mutation probabilities provided by the deep learning model could provide valuable information for further diagnosis and treatment.

The test set comprised 70 surgical specimens and 30 biopsies. We found that the sensitivity, specificity, and accuracy of the prediction model for surgical specimens were better than those for biopsies (81.6% vs. 58.3%, 78.7% vs. 66.7%, and 80.0% vs. 63.3%, respectively). This difference was mainly due to the small sample size of biopsies, leading to a small set of training data for the prediction model. In clinical workflows, EGFR mutation prediction needs to be performed on biopsies, since it is impossible to perform surgical operations for patients with advanced NSCLC. In future research, we will increase the amount of training data and improve the prediction accuracy for biopsy samples.

Among the 12 cases of SCC, 10 were correctly predicted (accuracy: 83.33%, specificity: 90.9%, NPV: 90.9%). For the two misclassified cases, we reviewed the H&E-stained slides and discovered several characteristics. For the false positive, the SCC was similar in structure to the papillary type (small cancer nests with obvious vascular axes). For the false negative, the tumour cell mass was small and damaged.

In addition to the EGFR mutation status of ADC [10], we investigated different types of lung cancer (ADC and SCC) and five subtypes of ADC. For 88 ADC cases, the sensitivity, specificity, PPV, and NPV of the prediction model were 77.6%, 69.2%, 76.0%, 71.1%, and 73.9%, respectively.

With the information on ADC subtypes, we were able to make improved accurate and detailed predictions. The sensitivity of the EGFR prediction model for ADC increased from 77.6% to 79.2%, and the specificity increased from 69.2% to 71.4%.

Current artificial intelligence systems can assist pathologists in diagnosing routine samples more efficiently, but they lack the ability to handle challenging cases. For these samples, it is more meaningful to submit them to the pathologist for a definitive diagnosis. Artificial intelligence serves pathologists rather than replacing them. We should focus on the combined effectiveness of humans and artificial intelligence, rather than solely on human improvement.

We applied the double-threshold to the entire NSCLC test set and each subtype of ADC. For the entire NSCLC test set, we set the double thresholds to 0.50 and 0.16 and achieved a sensitivity of 100.0% and a specificity of 87.5%. After stratification, the NSCLC EGFR mutation status could be determined from H&E-stained slides with high accuracy in 33% of cases. Sixty seven percent of the cases were between the two thresholds and needed to be confirmed by PCR/NGS.

For subtypes of ADC, the double-threshold filtered out 37.3% of the samples with 100% sensitivity. For the cases between the two thresholds, we reviewed the H&E-stained slides and did not find obvious visual characteristics.

This study is based on the lung cancer recognition model we established earlier, which used ADC, SCC, small cell carcinoma and normal lung tissue as labels. The lung cancer detection model could not identify subtypes of ADC. In this study, the dominant subtypes of ADC were determined by pathologists, which introduced a certain degree of subjectivity. In future work, we aim to build models using subtypes as additional training information.

One important limitation of this study pertains to the small sample size employed for training and testing the deep learning model. The dataset used in this study consisted of 326 participants, which may limit the generalizability of the deep learning model. In future work, we will address this limitation by conducting studies with a larger sample size obtained from multiple medical centers.

## Conclusion

In summary, the proposed EGFR mutation prediction model shows great promise for clinical application under circumstances where the sample is insufficient or the patient’s medical condition is poor. In pathological diagnosis, subjectivity and inconsistency exist in classifying ADC and estimating the cancerous area from WSIs. In future work, we will establish a combined framework to integrate diagnosis, quantitative analysis, and EGFR mutation prediction into one complete pipeline to enhance the model performance and clinical applicability.

## Data Availability

The data that support the findings of this study are available on request from the corresponding authors. The ResNet-50 model structure for EGFR mutation prediction was opensourced at: https://github.com/ThoroughFuture/EGFR. The pixel-level cancer detection model structure on large-scale pathological images was opensourced at: https://github.com/ThoroughFuture/PathFrame.

## Abbreviations

EGFR: Epidermal growth factor receptor
NSCLC: Non-small cell lung cancer
PCR: Polymerase chain reaction
NGS: Next-generation sequencing
WSI: Whole-slide image
ADC: Adenocarcinoma
AUC: Area under the curve
SCC: Squamous cell carcinomas
H&E: Hematoxylin and eosin
ROC: Receiver operating characteristic
PPV: Positive predict value
NPV: Negative predict value
